# Orchestration an extracellular lipase production from *Aspergillus niger* MYA 135: biomass morphology and fungal physiology

**DOI:** 10.1186/s13568-021-01202-y

**Published:** 2021-03-17

**Authors:** Hebe Natalia Salvatierra, Erika Lucía Regner, Mario Domingo Baigorí, Licia María Pera

**Affiliations:** 1 Morphogenesis and Fermentation Lab, PROIMI-CONICET, T4001 MVB San Miguel de Tucumán, Argentina; 2 Facultad de Bioquímica, Química y Farmacia, Cátedra de Microbiología Superior, Universidad Nacional de Tucumán, T4000INI San Miguel de Tucumán, Argentina

**Keywords:** Lipase production, *Aspergillus niger*, Biomass morphology, Fungal physiology

## Abstract

The impact of biomass morphology and culture conditions on fungal fermentation was widely reviewed in the literature. In this work, we presented three independent experiments in order to evaluate the influence of some of those input factors on a lipase production separately by using the *Aspergillus niger* MYA 135 and the two-stage fermentation technique. Regarding the culture modality, the biomass was pre-grown in a first reactor. Then, the washed mycelium was transferred to a second reactor to continue the study. Firstly, linear effects of fungal morphology and several physiological parameters on a lipase production were explored using the Plackett–Burman design. The dispersed fungal morphology was confirmed as a proper quality characteristic for producing an extracellular lipase activity. Concerning the impact of the carbon source on the biomass pre-growth, the sucrose (E = 9.923, *p* < 0.001) and the l-arabinose (E = 4.198, *p* = 0.009) presented positive and significant effects on the enzyme production. On the contrary, the supplementation of 0.05 g/L CaCl_2_ displayed a highly negative and significant effect on this process (E = − 7.390, *p* < 0.001). Secondly, the relationship between the enzyme production and the input variables N:C ratio, FeCl_3_ and olive oil was explored applying the central composite design. Among the model terms, the N:C ratio of the production medium had the most negative and significant influence on the enzyme synthesis. Thus, it was concluded that a low N:C ratio was preferable to increase its production. In addition, the bifunctional role of FeCl_3_ on this fungus was presented. Thirdly, a prove of concept assay was also discussed.

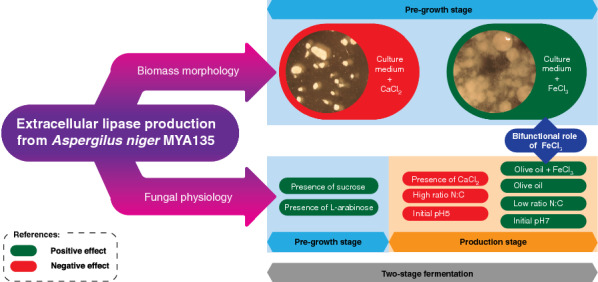

## Introduction

Lipases (EC 3.1.1.3) are versatile catalysts that have been used in hydrolytic and synthetic reactions (Verma et al. 2021). As shown in Table 1, a search made by our team covering academic and invention patent documents displayed an increasing interest of this enzyme in several industrial applications, with a significant growth over the last decade. Food and flavour industries were the most relevant sectors for products using lipases. In the second place, it can mention the number of total patent documents related to pharmaceutical industries and medical diagnostic. The oleochemical and waste-related lipase applications exhibited an accelerated growing during the last 10 years. In addition, the biosensor field was detected as an emerging category involving the utilization of this enzyme.

**Table 1:** General interest of lipase-related applications during the last four decades

Search methodology (field combinations)	Database									
	PubMed					Patentscope				
Lipase AND (lipase application combined with OR) AND Date of publication	Decade				Total	Decade				Total
	1980–1989	1990–1999	2000–2009	2010–2019	1980–2020	1980–1989	1990–1999	2000–2009	2010–2019	1980–2020

Lipase applications	Number of documents									
Pharmaceutical, Medical diagnostic	2	8	50	170	240	2	76	211	302	602
Oleochemical, Bio/fuel^a^	0	1	15	346	369	0	1	22	84	105
Agrochemical	0	0	0	4	5	0	4	1	3	8
Food, Flavour	233	289	616	1,299	2509	13	100	289	584	1035
Detergent	49	65	65	134	322	32	179	100	211	535
Biosensor	0	0	8	46	56	0	4	2	6	12
Waste, Valorization, Waste management, Bioremediation	2	20	127	354	529	2	18	58	153	233
Cosmetic	0	4	28	117	156	0	26	99	49	171

^a^To represent the scope of this article, the keyword *biofuel* was more appropriated in PubMed database; while, the keyword *fuel* was more appropriated in Patentscope database

Filamentous fungi as enzyme sources are widely used because they are able to produce a large amount of proteins. Native or recombinant biocatalysts from *Aspergillus niger*, *A. oryzae* and *Trichoderma reesei* have been frequently reported. As an example, a lipase from *A. niger* was immobilized onto a novel macroporous acrylic resin getting a low-cost, stable and recyclable biocatalyst for deacidification of high-acid soy sauce residue oil (Feng et al. 2020). However, fermentations involving these complexes microorganisms are affected by the interrelation of several parameters, including biomass morphology and culture conditions. In submerged fermentation, filamentous fungi displayed a dispersed (freely hyphae or lax clumps) or a pelletized (spherical agglomerates of hyphae) morphology (Quintanilla et al. 2015). Besides, to quantitatively measure the effect of culture conditions on fungal growth, particle parameters such as projected area, circularity, aspect ratio and surface roughness have been described to generate a dimensionless morphology number. Thus, both fructofuranosidase and glucoamylase production by *A. niger* has been negatively correlated with an increasing the morphological number; while, a hypothetical correlation between the morphological number and productivity is proposed for citric acid and secondary metabolites (Cairns et al. 2019). In the same way, the enzyme productions are influenced by physico-chemical factors that should be optimized to reach a maximum yield (Geoffry and Achur 2018a).

In other to control fungal morphology, several strategies have been reported. The conventional procedure is based on the adjustments of chemical, physical and biological parameters such as conidia concentration, medium composition, temperature, pH, supplementation of glass beads, agitation systems, fermenter geometry, etc. (Papagianni et al. 2004). Furthermore, Wucherpfennig et al. (2011) reported that the culture osmolality also affects fungal morphology and process productivity. In another approach, the macroscopic fungal morphology was tailoring by altering the hyphal morphology and the conidia adhesion capacity (Colin et al. 2013). More recently, Karahalil et al. (2019) reviewed several aspects of an interesting technique named microparticle enhanced cultivation. That method allows the control of growth physically in submerged fermentation by blocking the aggregation of filamentous microorganisms using microparticles such as talc, aluminium oxide, titanium silicom oxide, iron (II, III) and forsterite.

Thus, as the biomass morphology and the culture conditions can affect the process productivity, the influence of those variables should be study separately. To do that, the two-stage fermentation strategy can be used being the replacement technique one of this culture modality (Sternberg and Mandels 1979). Briefly, the biomass is pre-grown in a first reactor. Then, the washed mycelium is transferred to a second reactor to continue the study. For instance, this approach was reported to analyse the transcriptional regulation of *xyn*2 in *Hypocrea jecorina* (Würleitner et al. 2003).

In this work, the main objective was to study the effect of biomass morphology and physiological factors on an extracellular lipase production from *Aspergillus niger* MYA 135 by using a submerged two-stage fermentation.

## Material and methods

### Microorganism and culture conditions

The native microorganism *Aspergillus niger* ATCC MYA 135, formerly known as *A. niger* 419 from our culture collection (PROIMI-CONICET), was used throughout this work. The strain was maintained by monthly transfer onto glucose potato agar slants, incubated at 30 °C and stored at 4 °C. Submerged fermentation was also carried out at 30 °C on an orbital shaker (INFORS) at 200 rpm. Flasks were inoculated with a conidial suspension to get a final concentration of about 10^5^ conidia m/L.

### Biomass determination

The biomass was estimated by drying washed mycelia at 105 °C until constant weight (Colin et al. 2013).

### Protein determination and native PAGE

Protein concentration was determined according to Bradford (1976). Additionally, proteins were separated by native-PAGE using 10% (by mass per volume) polyacrylamide gel. The presence of a lipolytic band was detected using 1.3 mM of α-naphtyl acetate as substrate. Released naphthol was coupled with 1 mM Fast Blue to give a coloured product. Reactions were carried out at 37 °C in shaken plates containing 100 mM phosphate buffer (pH = 7.0).

### Lipase determination

The lipase activity was measured according to Winkler and Stuckman (1979). One unit of enzyme activity was defined as the amount of enzyme that released 1 μmol of *p*-nitro phenol per min. Lipase production was expressed either as a volumetric activity (units per liter of culture supernatant, U/L) or a specific activity (units per gram of biomass dry weight, U/g_dry weight_; units per milligram of protein, U/mg_protein_).

### Scanning electron microscopy

For observation with scanning electron microscope (SEM), at the end of fermentation, the mycelium was collected by filtration, washed with 0.1 mM phosphate buffer (pH 7), fixed with 2.5% glutaraldehyde and postfixed with 1% OsO_4_. The mycelium was dehydrated in acetone, dried in a critical point apparatus, coated with gold and observed by using a Zeiss Supra 55VP (Carl Zeiss, Oberkochen, Germany).

### Two-stage lipase production: replacement technique

Assays were conducted in 50 mL conical flasks containing 10 mL of culture medium. The use of this working volume was previously reported for an extracellular lipase production by *A. niger* MYA 135 (Colin et al. 2010). For evaluate both biomass morphology and physiological effectors on the lipase production, the replacement technique reported by Sternberg and Mandels (1979) was used. Briefly, mycelia were pre-grown during 24 h, at initial pH 5 and in the presence of 2 g/L NH_4_NO_3_. In the case of Plackett–Burman experimental design, the pre-growth fungal stage was conducted using morphological inducers and carbon sources detailed in Table 2. To induce pelleted or dispersed fungal morphology the corresponding culture medium was supplemented with 0.5 g/L CaCl_2_ or 1.0 g/L FeCl_3_, respectively (Colin et al. 2013). In the case of central composite design, the pre-growth fungal stage was conducted under a unique condition using 1 g/L FeCl_3_ and 10 g/L sucrose as morphological inducer and carbon source, respectively. Biomasses were collected and washed by vacuum filtration; then, they were weighted and added to different lipase production culture media according to the experimental design (Plackett–Burman experimental design or central composite design). The initial biomass concentration was 8 and 15 g_wet weight_/L for Plackett–Burman and central composite experimental designs, respectively. Flasks were further incubated for 72 h under the same conditions. Both biomass pre-growth media and lipase production media contained the same inorganic supplement (in g/L): KH_2_PO_4_ 1.0, MgSO_4_7H_2_O 0.2, CuSO_4_5H_2_O 0.06. The nitrogen:carbon (N:C) ratio was studied in production media by varying the concentration of NH_4_NO_3_ keeping the concentration of sucrose at 5 g/L.The supernatant was obtained by vacuum filtration and used as enzyme source.

**Table 2:** Plackett–Burman design: experimental procedure, variables and statistical analysis

Two-stage Fermentation	Pre-growth stage (24 h) Initial pH 5				Lipase production stage (72 h) Initial biomass concentration: 8 g_wet weight_ /L							Response variable
Input variables	**Morphology (−) pellets (+) dispersed**	**Glucose**	**l**-**Arabinose**	**Sucrose**	N:C ratio	pH	Oleic acid	Tributirine	CaCl_2_	FeCl_3_	Olive oil	Specific lipase activity (U/g_dry weight_)
Level (g/L)^a^												
(−)	**0.5 CaCl**_**2**_	**0**	**0**	**5.0**	0.4	5.0	0	0	0	0	10.0	
(+)	**1.0 FeCl**_**3**_	**10.0**	**10.0**	**10.0**	0.6	7.0	1.0	1.0	0.05	0.1	20.0	
Trial number												
1	+	−	+	+	−	+	−	−	−	+	+	33.92 ± 1.53
2	−	−	+	+	+	−	+	+	−	+	−	17.32 ± 3.72
3	−	−	−	+	+	+	−	+	+	−	+	17.88 ± 3.22
4	+	+	−	+	−	−	−	+	+	+	−	11.84 ± 1.94
5	−	+	−	−	−	+	+	+	−	+	+	15.33 ± 0.04
6	+	−	−	−	+	+	+	−	+	+	−	7.61 ± 0.28
7	−	−	−	−	−	−	−	−	−	−	−	3.12 ± 0.07
8	−	+	+	+	−	+	+	−	+	−	−	17.19 ± 3.68
9	+	+	−	+	+	−	+	−	−	−	+	24.47 ± 6.66
10	−	+	+	−	+	−	−	−	+	+	−	11.41 ± 1.51
11	+	+	+	−	+	+	−	+	−	−	−	20.84 ± 5.88
12	+	−	+	−	−	−	+	+	+	−	+	4.75 ± 1.81
Effect	3.531	2.747	4.198	9.923	2.232	6.643	− 2.053	− 1.625	− 7.390	1.530	3.978	R^2^ = 92.73%
*p*-value	**0.022**	**0.062**	**0.009**	**< 0.001**	0.121	< 0.001	0.151	0.248	< 0.001	0.275	0.003	R^2^_adj_ = 86.07%

Letters in bold show the experimental procedure, variables and statistical analysis corresponding to the pre-growth stage

^a^Concentration of medium components

### Two-stage lipase production 1: Plackett–Burman experimental design

The effect of 11 environmental factors on the enzyme production expressed as lipase units per gram of biomass dry weight (U/g_dry weight_) were evaluated after 96 h of cultivation by using the Plackett–Burman experimental design (Plackett and Burman 1946) (Table 2). This is a two-level fractional factorial design for studying *n*-1 input variables (factors) using *n* runs, where *n* is a multiple of 4 (*n* is the number of experiments). Each factor is represented at two levels, high and low, which are denoted by (+) and (−), respectively. The effect for a factor is always described as the change in the response in going from the low level of that factor to the high level. A negative sign means that going from low level to high level for a factor decreases the response. A positive sign means that going from the low level to the high level increases the response. The effect (E) of each variable on the response was determined by subtracting the average response of the low level from that of the high level. Levels of each input variable were decided based on either our own experience or literature reports (Pokorny et al. 1994; Gordillo et al. 1995; Colin et al. 2010; Griebeler et al. 2011; Lanka et al. 2015). The experimental error was determined by replication of the entire experimental matrix.

### Two-stage lipase production 2: central composite design

The relationship between the three input variables N:C ratio, FeCl_3_ and olive oil, and the enzyme production expressed as lipase units per liter of culture supernatant (U/L) were evaluated after 96 h of cultivation by using the central composite design (Myers and Montgomery 2002). This is an experimental design for building a second order polynomial for the response variable. It involves three types of trials: 2* k* factorial trials, 2* k* axial trials and *n*c center point trials, where *k* is number of factors studied in the assay. Values at center point provide information about the existence of curvature in the response; that is, they contribute to the estimation of quadratic terms. Axial points are also used to estimate quadratic terms, while factorial points contribute to the estimation of linear and interaction terms. Each factor was studied at five different levels (− α, − 1,0 , + 1, + α) (Table 3). Variable settings that represent the axial point of the design were decided based on either our own experience or literature reports (Pokorny et al. 1994; Colin et al. 2010; Salihu et al. 2011). The experimental error was determined by replication of the entire experimental matrix.

**Table 3:** Central composite design: input variables and their levels

Input variables	Levels				
	− α	− 1	0	+ 1	+ α
X_1_					
N:C ratio	0.200	0.281	0.400	0.519	0.600
X_2_					
FeCl_3_ (g/L)	0.050	0.080	0.125	0.170	0.200
X_3_					
Olive oil (g/L)	10.000	14.000	20.000	26.000	30.000

### Two-stage lipase production 3: a prove of concept

The assay was conducted in 500 mL conical flasks containing 100 mL of culture medium. The fermentation medium comprised (in g/L): sucrose 10.0, KH_2_PO_4_ 1.0, NH_4_NO_3_ 2.0, MgSO_4_ 7H_2_O 2.0, CuSO_4_ 0.06 and FeCl_3_ 1.0. The initial pH was adjusted to 7.0 with NaOH. After 24 h of incubation, the culture was transferred to another 500 mL flask containing 50 mL of 3% (by volume) and was further incubated for 168 h under the same conditions. The lipase production was monitored during fermentation using as response variables either the enzyme activity expressed as lipase units per liter of culture supernatant (U/L) or the specific lipase activity expressed as enzyme units per milligram of protein (U/mg_protein_).

### Statistical analysis

All statistical analysis was performed using Minitab software (Minitab Inc., State College, PA, USA). Data were expressed as means ± standard deviation. Differences were accepted as significant when *p* < 0.05. The fitness of models was checked by both the determination coefficient (R^2^) and the adjusted determination coefficient (adj R^2^).

## Results

Previously, it was reported an olive oil-induced extracellular lipase activity by the native *A. niger* MYA 135 using a mineral culture medium (Colin et al. 2010). In this work, the employ of statistically designed experiments was proposed to obtain knowledge about the relationship among some input factors and the production of this extracellular enzyme. The results of the three two-stage fermentations are described below.

### Two-stage lipase production 1: Plackett–Burman experimental design

Firstly, linear effects of fungal morphology and several physiological parameters on a lipase activity production were explored using a fractional experimental design. As both fungal morphology and physiology are affected by the same environmental conditions, the biomass and the lipase production was uncouple using the replacement technique. Considering that our main objective in this assay was to evaluate the impact of biomass morphology on the lipase activity production, it was decided to determine the enzyme activity expressed as U/g_dry weight_. Table 2 shows the Plackett- Burman design for 12 trials and the corresponding response variable. The R^2^ value indicated that 92.73% of data variation was explained by the input variables. The adj R^2^ was 86.07%. Thus, data on specific enzyme activity exhibited a wide variation covering the range from 3.12 ± 0.07 to 33.92 ± 1.53 U/g_dry weight_. The fungal morphology displayed a significant effect on the final response being the dispersed mycelia more favorable than the pelleted form of growth for to increase the specific lipase activity. Concerning the impact of the carbon source on the biomass pre-growth, both the sucrose and the l-arabinose presented positive and significant effects on the enzyme production, while the effect of glucose was not significant under our assay conditions. In relation to the effect of physiological parameters on the lipase production, as expected, the olive oil had a positive and a significant effect on the lipase activity being negligible the presence of either oleic acid or tributyrin. Besides, the initial pH 7 of culture medium was more favorable to the enzyme production than the initial pH 5. Regarding the influence of ions, the supplementation of 0.05 g/L CaCl_2_ displayed a highly negative and significant effect on the enzyme production (E = − 7.390). The linear effect corresponding to the input variable FeCl_3_ was not significant (p = 0.275).

### Two-stage lipase production 2: central composite design

Considering that our next objective was to explore the relationship between the enzyme production and the input variables N:C ratio (X_1_), FeCl_3_ (X_2_) and olive oil (X_3_), it was decided to apply the central composite design for 20 trials expressing the enzyme activity as U/L. As it was mentioned before, with this kind of experimental design is possible to estimate linear, quadratic and interaction effects. To conduct this experiment, the biomass was developed in the presence of 1 g/L FeCl_3_; and then, it was collected, washed, weighted and added to different lipase production media according to the experimental design (Table 4). Data on enzyme activity showed a wide variation covering the range from 1.57 ± 0.41 to 324.57 ± 19.86 U/L. Considering the *p*-value of the corresponding coefficients (0.007 or smaller), the lipase activity production depended on two linear effects (X_1_ and X_3_), one two-way interaction (X_2_X_3_) and two quadratic effects (X_1_^2^ and X_2_^2^) (Table 5). The second-order polynomial equation is shown below:

$$\begin{aligned} {\text{Y}}_{{({\text{U}}/{\text{L}})}} & = {195}.0{-}{61}.9{\text{X}}_{{1}} + {48}.8{\text{X}}_{{3}} + {39}.8{\text{X}}_{{2}} {\text{X}}_{{3}} \\ & \quad {-}{64}.3{\text{X}}_{{1}}^{{2}} + {3}0.7{\text{X}}_{{3}}^{{2}} \end{aligned}$$

**Table 4:** Central composite design: experimental procedure and variables

Two-stage fermentation				
Pre-growth stage (24 h) in the presense of 1.0 g/L FeCl_3_ Biomass morphology: dispersed mycelium				
Lipase production stage (72 h), initial biomass concentration: 15 g_wet weight_/L				
Trial number	Input variables			Response variable
	N:C ratio	FeCl_3_	Olive oil	Lipase activity (U/L)
CP	0	0	0	153.73 ± 2.17
CP	0	0	0	82,94 ± 0.29
CP	0	0	0	219.61 ± 6.50
CP	0	0	0	191.01 ± 5.78
CP	0	0	0	238.76 ± 14.80
CP	0	0	0	239.78 ± 5.42
1	+ 1	+ 1	− 1	3.33 ± 0.21
2	− 1	− 1	+ 1	294.90 ± 25.94
3	0	0	− α	89.59 ± 1.73
4	− 1	+ 1	+ 1	324.57 ± 19.86
5	+ 1	− 1	− 1	20.13 ± 0.87
6	+ 1	− 1	+ 1	6.56 ± 4.27
7	0	+ α	0	315.63 ± 32.50
8	0	0	+ α	251.28 ± 10.47
9	− 1	+ 1	− 1	135.60 ± 10.11
10	+ 1	+ 1	+ 1	170.84 ± 7.95
11	0	− α	0	276.05 ± 48.76
12	+ α	0	0	1.57 ± 0.41
13	− α	0	0	30.16 ± 11.66
14	− 1	− 1	− 1	242.98 ± 30.52

**Table 5:** Central composite design: terms and the corresponding coefficients

Term	Coefficient	*p*-value
Constant	195.0	< 0.001
Linear		< 0.001
X_1_ (N:C ratio)	− 61.9	< 0.001
X_2_ (FeCl_3_)	12.7	0.251
X_3_ (Olive oil)	48.8	< 0.001
Interaction		< 0.001
X_1_X_2_	28.1	0.057
X_1_X_3_	− 10.9	0.450
X_2_X_3_	39.8	0.009
Quadratic		0.015
X_1_^2^	− 64.3	< 0.001
X_2_^2^	30.7	0.007
X_3_^2^	− 9.7	0.368
Lack-of-fit		0.578
R^2^ (%)	80.10	
R^2^_adj_ (%)	73.24	

As it can be seen, both linear and quadratic terms corresponding to the N:C ratio were negative. While, those terms associated to olive oil as well as its interaction with FeCl_3_ were positive. In addition, the R^2^ value indicated that 80.10% of data variation was explained by the input variables. The adj R^2^ was 73.24%. The lack-of-fit test is testing the lack of fit for the quadratic model; the *p*-value for this test is large (*p* = 0.578) implying that the quadratic model was adequate (Table 5).

### Two-stage lipase production 3: a prove of concept

**Fig. 1:**
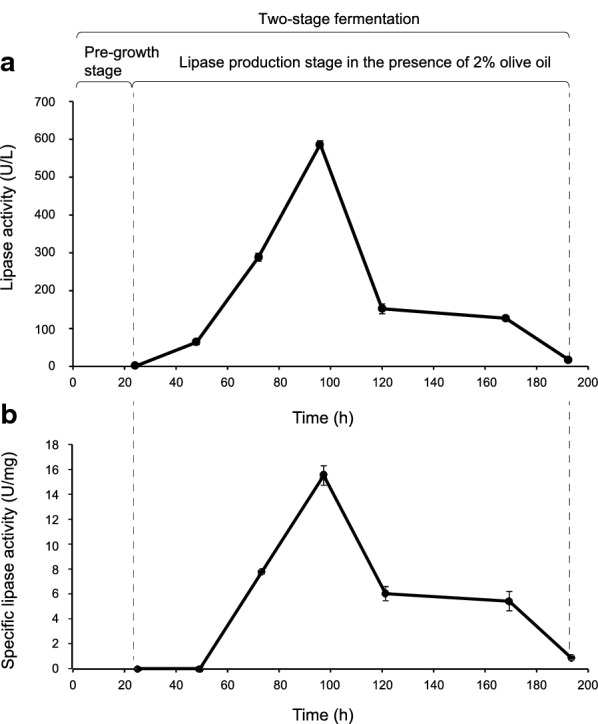
Time course of an extracellular lipase activity from *Aspergillus niger* MYA 135 during the lipase production stage expressed as volumetric activity (U/L) (**a**) or specific activity (U/mg_protein_) (**b**)

**Fig. 2:**
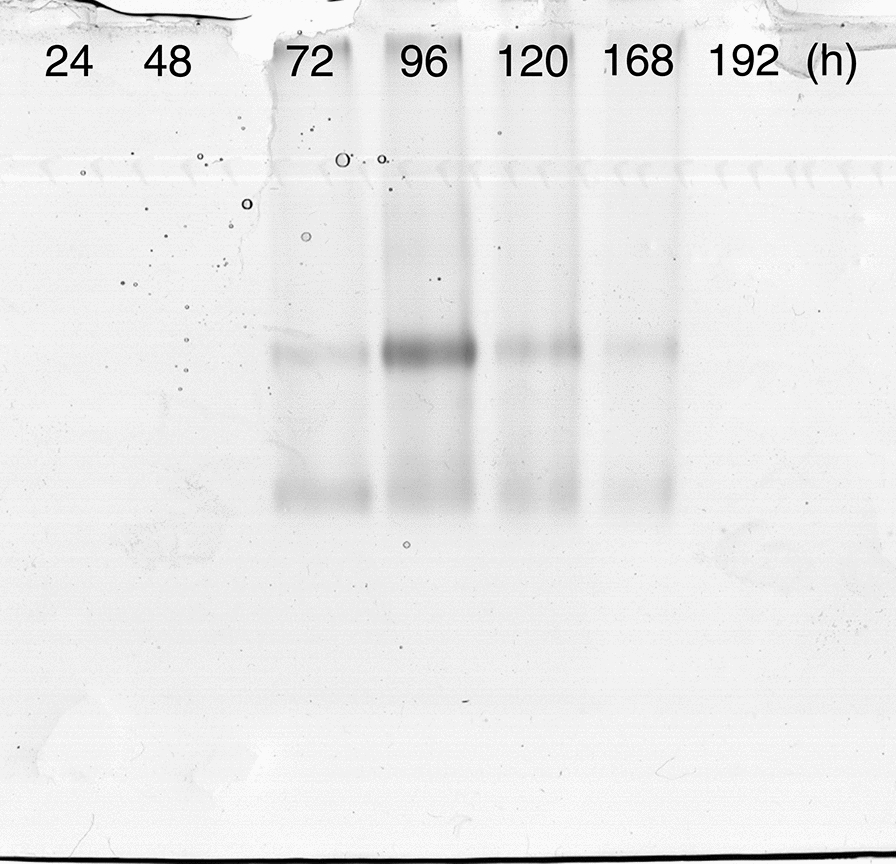
Native PAGE showing the time course of an extracellular lipase activity from *Aspergillus niger* MYA 135 during the lipase production stage. The lipolytic bands were detected using α-naphtyl acetate as substrate

**Fig. 3:**
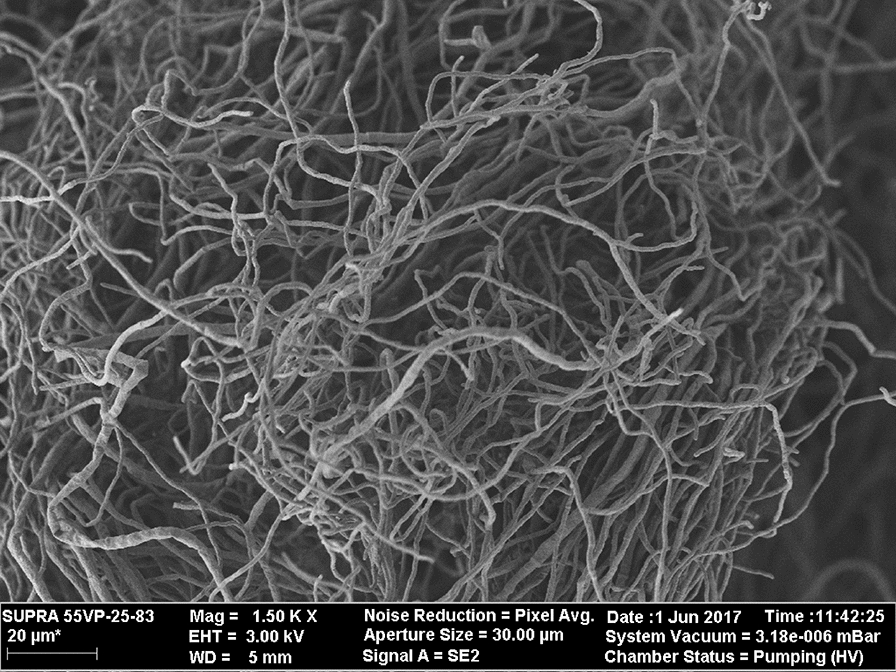
Image of dispersed mycelium obtained with a scanning electron microscope showing the harvested biomass from *Aspergillus niger* MYA 135 after 96 h of cultivation

Taking in mind the results obtained, a third two-stage lipase production was conducted as a prove of concept. The biomass was pre-grown at an initial pH 7, in the presence of 1 g/L FeCl_3_ and with a starting N:C ratio of 0.2 as described in the material and methods section. After 24 h of cultivation, the entire culture was transferred to another reactor without any washed procedure getting an initial olive oil concentration of 20 g/L. The highest value of lipase activity (U/L) (Fig. 1a) and specific lipase activity (U/mg_protein_) (Fig. 1b) was obtained after 96 h of cultivation. In addition, the culture supernatant was analyzed by native PAGE. Two lipolytic bands were detected using α-naphtyl acetate as substrate being the intensity signal of the top one also increased at the time of 96 h (Fig. 2). On the other hand, SEM micrographs of the harvested biomass displayed a dispersed mycelium (lax clumps mixed with free mycelium) showing scarcely branched and not fragmentated hyphae; in addition, the presence of conidiophores was not observed (Fig. 3).

## Discussion

The impact of biomass morphology and culture conditions on fungal fermentation was analyzed in several investigations as it was briefly mentioned in “Introduction” section.

Here, through the application of designed experiments, we evaluated the influence of some of those input factors on a lipase activity production separately by using the *A. niger* MYA 135 as a model and the two-stage fermentation technique as the culture modality.

### Biomass morphology and lipase production

In coincidence with the results reviewed by Krull et al. (2010), the macroscopic characteristic of fungal morphology displaying by *A. niger* MYA 135 was already apparent after about 18 h of incubation (data not shown). For that reason, it was decided to conduct a biomass pre-growth stage of 24 h. So, culture media at initial pH 5 were supplemented with morphological inducers in the pre-growth fermentation stage. As previously reported, dispersed mycelium is induced in the presence of 1 g/L FeCl_3_; while, the pelleted form of growth is favoured by the addition of 0.5 g/L CaCl_2_ (Colin et al. 2013). In addition, under our culture conditions, before that time of cultivation, no significant extracellular lipase activity was found (data not shown). Thus, the influence of the biomass morphology on the specific lipase production expressed as U/g_dry weight_ was, firstly, evaluated. Dispersed or pelleted mycelia were harvested, washed by vacuum filtration, weighted as indicated in the material and methods section, and then added to different production media. The Plackett–Burman experimental design identified the significant effect of the biomass morphology being the dispersed mycelium preferred to increase the specific lipase production. In fact, the same culture medium at an initial pH 7 without FeCl_3_ supplementation favours the pelleted form of growth; and, under that environmental condition, the maximum specific activity using *p*-NPP as substrate was 1.6 U/mg_protein_ after 96 h of cultivation (Pera et al. 2006). Here, as it can be seen in the prove of concept assay, that value was increased ten times in the presence of dispersed mycelium (Fig. 1b). In concordance with our observation, a freely dispersed mycelium from *A. niger* SKAn1015 induced by the supplementation of silicate microparticles was linked to an increase of a fructofuranosidase activity displaying the microparticles itself an important physiological role (Driouch et al. 2010). On the contrary, the performance of a synthetic mycelium-bound lipase activity from *Rhizopus chinensis* was favoured by fully entangled mycelial filaments (Teng et al. 2009). However, as it will be discussed later, the lipase production by filamentous fungi should be analyzed from a holistic point of view.

### Fungal physiology and lipase production

Firstly, the impact of several physiological factors on the lipase production activity expressed as U/g_dry weight_ (Two-stage lipase production 1) was evaluated. Thus, besides the fungal biomass morphology, the kind of carbon source using during the pre-growth stage also had a relevant role on the specific lipase production. In fact, according to the Plackett–Burman experimental design, the presence of sucrose during the first-stage of fermentation exhibited the most significant and positive effect on the response variable. On the other hand, the olive oil concentration also displayed a positive effect on the response variable being the effect associated with the supplementation of either oleic acid or tributyrin not significant. So, the hydrophilic substrate could have a relevant role during the fungal growth stage, while the hydrophobic one acts mainly as an inducer of the lipolytic activity synthesis. These results were compatible with those that reported the use of a combination of carbohydrate source and lipid inducer as a strategy to increase the biocatalyst production. However, the proper mixture of those culture components varies according to the microorganism used. To give examples, the optimal substrates for a lipase activity production from *Pichia lynferdii* Y-7723 were soybean oil and sucrose (Kim et al. 2010); while, olive oil and xylose were preferred by *Rhizopus oryzae* ZAC3 (Ayinla et al. 2017). Concerning *A. niger* MYA 135, the olive oil was the best hydrophobic substrate using in the presence of sucrose (Colin et al. 2010). Additionally, as it was also shown in this work, the initial pH value of the production medium is an important physiological factor involved in the lipase production being this parameter a fungi-dependent variable. In this connection, it was reported the production of fungal lipases under acidic (Turati et al. 2019), near neutral (Colla et al. 2016) or alkaline (Rajan and Nair 2011) culture conditions. Finally, the impact of metal ions on the lipolytic enzyme synthesis has been evaluated in several studies during the selection of significant input variables (Rajendran and Thangavelu 2009; Salihu et al. 2011). In this work, the supplementation of 0.1 g/L FeCl_3_ did not has a significant effect on the specific enzyme activity; other aspects concerning to this salt are discussed in the next paragraph. On the contrary, the presence of 0.05 g/L CaCl_2_ exhibited a highly negative impact on the response (E = − 7.390). Similarly, Geoffry and Achur (2018a), reported that the variable CaCl_2_ influences negatively on the production of a lipase activity from *Fusarium solani*. Oppositely, the supplementation of this salt is favorable for the synthesis of a lipase activity using *Rhizopus arrhizus* MTCC 2233 (Rajendran and Thangavelu 2009). And, the effect of CaCl_2_ is no significant to produce a lipase activity from *Yarrowia lipolytica* MTCC 35 (Kishan et al. 2013). Thus, reported data clearly indicate the wide variation in effects of CaCl_2_ on the lipase production.

Secondly, the central composite design was adopted to study the relationship between the lipase production and the input variables N:C ratio, FeCl_3_ and olive oil. In this case, the response variable was the volumetric enzyme activity expressed as U/L. This parameter was measured after 96 h of cultivation (Two-stage lipase production 2). Concerning the effect of nitrogen source on the lipase production, there is a general agreement in the literature that this enzyme synthesis is favoured at high nitrogen concentration, and therefore, lower C:N ratios, being this substrate either organic or inorganic (Das et al. 2016; Geoffry and Achur 2018b). In this connection, Coradi et al. (2013) reported that *Trichoderma harzianum* displayed the highest lipase activity in a culture medium containing 0.5% yeast extract and 1% olive oil (2.5 C:N ratio). Here, among the model terms, the N:C ratio of the production culture medium had the most significant influence on the lipase activity production. A negative linear coefficient suggests that as the N:C ratio increases, the independent variable tends to decrease. In addition, as the corresponding quadratic term was also negative, the net effect of increasing the N:C ratio was an acceleration of the response variable decrease. Thus, by using the replacement technique it was demonstrated that a low N:C ratio was preferable to increase the lipase production by *A. niger* MYA 135. This effect was also observed in the prove of concept assay where the initial N:C ratio was 0.2 (or 5 C:N ratio). As expected, the next important factor was the olive oil concentration. Not only the linear term corresponding to this input variable was positive (p < 0.001) but also a positive interaction between olive oil and FeCl_3_ (p = 0.009) was found. Thus, as the linear term corresponding to olive oil was positive, the effect on the response will tend to be more positive increasing the value of FeCl_3_ concentration. Thus, the FeCl_3_ acted not only as a morphological effector but also as an additive that contributed to increase the lipase production. However, according to ours results, the N:C ratio always has to be taken into account when evaluating the impact of other input variables on the lipase activity.

Finally, the dispersed mycelium produced at least two lipases as it was observed in native PAGE being the intensity signal of the top one increased after 72 h of incubation. In addition, the lipase production pattern of *A. niger* MYA 135 was similar to those reported for *Aspergillus carbonarius* (Ire and Ike 2014) and *Rhizopus oryzae* ZAC3 (Ayinla et al. 2017) achieving all of them an optimum incubation time of 96 h.

In summary, analysing the three independent assays conducted during this work, it can be highlighted the usefulness of the replacement technique to study how environmental conditions affected a lipase activity production evaluating the influence of biomass morphology and other physiological effectors separately. The dispersed morphology was confirmed as a proper quality characteristic for producing a lipase activity from *A. niger* MYA 135. In addition, the bifunctional role of FeCl_3_ on this fungus was presented. Finally, according to ours results, the N:C ratio always has to be taken into account when evaluating the impact of other input variables on this process using this filamentous fungus.

## Data Availability

Authors can confirm that all relevant data are included in the article.

## References

[CR1] Ayinla ZA, Ademakinwa AN, Agboola FK (2017). Studies on the optimization of lipase production by *Rhizopus *sp. ZAC3 isolated from the contaminated soil of a palm oil processing shed. J App Biol Biotechnol.

[CR2] Bradford MM (1976). A rapid and sensitive method for the quantification of microgram quantities of protein utilizing the principle of protein-dye binding. Anal Biochem.

[CR3] Cairns TC, Zheng X, Zheng P, Sun J, Meyer V (2019). Moulding the mould: understanding and reprogramming filamentous fungal growth and morphogenesis for next generation cell factories. Biotechnol Biofuels.

[CR4] Colin VL, Baigori MD, Pera LM (2010). Effect of environmental conditions on extracellular lipases production and fungal morphology from *Aspergillus niger* MYA 135. J Basic Microbiol.

[CR5] Colin VL, Baigori MD, Pera LM (2013). Tailoring fungal morphology of *Aspergillus niger* MYA 135 by altering the hyphal morphology and the conidia adhesion capacity: biotechnological applications. AMB Expr.

[CR6] Colla LM, Primaz AL, Benedetti S, Loss RA, Lima M, Reinehr CO, Bertolin TE, Vieira Costa JA (2016). Surface response methodology for the optimization of lipase production under submerged fermentation by filamentous fungi. Braz J Microbiol.

[CR7] Coradi GV, da Visitação VL, de Lima EA, Saito LYT, Palmieri DA, Takita MA, Neto PO, Gomes de Lima VM (2013). Comparing submerged and solid-state fermentation of agro-industrial residues for the production and characterization of lipase by *Trichoderma harzianum*. Ann Microbiol.

[CR8] Das A, Bhattacharya S, Shivakumar S, Shakya S, Sogane SS (2016). Coconut oil induced production of a surfactant-compatible lipase from *Aspergillus tamarii* under submerged fermentation. J Basic Microbiol.

[CR9] Driouch H, Sommer B, Wittmann C (2010). Morphology engineering of *Aspergillus niger* for improved enzyme production. Biotechnol Bioeng.

[CR10] Feng K, Huang Z, Peng B, Dai W, Li Y, Zhu X, Chen Y, Tong X, Lan Y, Cao Y (2020). Immobilization of *Aspergillus niger* lipase onto a novel macroporous acrylic resin: Stable and recyclable biocatalysis for deacidification of high-acid soy sauce residue oil. Bioresour Technol.

[CR11] Geoffry K, Achur RN (2018). Optimization of novel halophilic lipase production by *Fusarium solani* strain NFCCL 4084 using palm oil mill effluent. J Genet Eng Biotechnol.

[CR12] Geoffry K, Achur RN (2018). Screening and production of lipase from fungal organisms. Biocatal Agric Biotechnol.

[CR13] Gordillo MA, Obradors N, Montesinos JL, Valero F, Lafuente J, Solà C (1995). Stability studies and effect of the initial oleic acid concentration on lipase production by *Candida rugose*. Appl Microbiol Biotechnol.

[CR14] Griebeler N, Polloni AE, Remonatto D, Arber F, Vardanega R, Cechet JL, Di Luccio M, de Oliveira D, Treichel H, Cansian RL, Rigo E, Ninow JL (2011). Isolation and screening of lipase-producing fungi with hydrolytic activity. Food Bioprocess Technol.

[CR15] Ire FS, Ike VC (2014). Screening and optimization of process parameters for the production of lipase in submerged fermentation by *Aspergillus carbonarius* (Bainer) IMI 366159. Annu Res Rev Biol.

[CR16] Karahalil E, Coban HB, Turhan I (2019). A current approach to the control of filamentous fungal growth in media: microparticle enhanced cultivation technique. Crit Rev Biotechnol.

[CR17] Kim HR, Kim IH, Hou CT, Kwon KI, SHIN BS, (2010). Production of a novel cold-active lipase from *Pichia lynferdii* Y-7723. J Agric Food Chem.

[CR18] Kishan G, Gopalakannan P, Muthukumaran C, Thirumalai Muthukumaresan K, Dharmendira Kumar M, Tamilarasan K (2013). Statistical optimization of critical medium components for lipase production from *Yarrowia lipolytica* (MTCC 35). J Genet Eng Biotechnol.

[CR19] Krull R, Cordes C, Horn H, Kampen I, Kwade A, Neu TR, Nörtemann B (2010). Morphology of filamentous fungi: linking cellular biology to process engineering using *Aspergillus niger*. Adv Biochem Eng Biotechnol.

[CR20] Lanka S, Pydipalli M, Latha JNL (2015). Optimization of process variables for extracellular lipase production from *Emericella nidulans* NFCCI 3643 isolated from palm oil mill effluent (POME) dump sites using OFAT method. Res J Microbiol.

[CR21] Myers R, Montgomery D (2002). Response surface methodology. Process and product optimization using designed experiments.

[CR22] Papagianni M (2004). Fungal morphology and metabolite production in submerged mycelial processes. Biotechnol Adv.

[CR23] Pera LM, Romero CM, Baigori MD, Castro GR (2006). Catalytic properties of lipase extracts from *Aspergillus niger*. Food Technol Biotechnol.

[CR24] Plackett RL, Burman JP (1946). Trust the design of optimum multifactorial experiments. Biometrika.

[CR25] Pokorny D, Friedrich J, Cimerman A (1994). Effect of nutritional factors on lipase biosynthesis by *Aspergillus niger*. Biotechnol Lett.

[CR26] Quintanilla D, Hagemann T, Hansen K, Gernaey KV (2015). Fungal morphology in industrial enzyme production—modelling and monitoring. Adv Biochem Eng Biotechnol.

[CR27] Rajan A, Nair AJ (2011). A comparative study on alkaline lipase production by a newly isolated *Aspergillus fumigatus* MTCC 9657 in submerged and solid-state fermentation using economically and industrially feasible substrate. Turk J Biol.

[CR28] Rajendran A, Thangavelu V (2009). Statistical experimental design for evaluation of medium components for lipase production by *Rhizopus arrhizus* MTCC 2233. LWT-Food Sci Technol.

[CR29] Salihu A, Alam MDZ, AbdulKarim MI, Salleh HM (2011). Optimization of lipase production by *Candida cylindracea* in palm oil mill effluent based medium using statistical experimental design. J Mol Catal B-Enzym.

[CR30] Sternberg D, Mandels GR (1979). Induction of cellulolytic enzymes in *Trichoderma reesei* by sophorose. J Bacteriol.

[CR31] Teng Y, Xu Y, Wang D (2009). Changes in morphology of *Rhizopus chinensis* in submerged fermentation and their effect on production of mycelium-bound lipase. Bioprocess Biosyst Eng.

[CR32] Turati DFM, Almeida AF, Terrone CC, Nascimento JMF, Terrasan CRF, Fernandez-Lorente G, Pessela BC, Guisan JM, Carmona EC (2019). Thermotolerant lipase from *Penicillium* sp. Section *Gracilenta* CBMAI 1583: effect of carbon sources on enzyme production, biochemical properties of crude and purified enzyme and substrate specificity. Biocatal Agric Biotechnol.

[CR33] Verma S, Meghwanshi GK, Kumar R (2021). Current perspectives for microbial lipases from extremophiles and metagenomics. Biochimie.

[CR34] Winkler UK, Stuckmann M (1979). Glycogen, hyaluronate, and some other polysaccharides greatly enhance the formation of exolipase by *Serratia marcescens*. J Bacteriol.

[CR35] Wucherpfennig T, Hestler T, Krull R (2011). Morphology engineering—osmolality and its effect on *Aspergillus niger* morphology and productivity. Microb Cell Fact.

[CR36] Würleitner E, Pera L, Wacenovsky C, Cziferszky A, Zeilinger S, Kubicek CP, Mach RL (2003). Transcriptional regulation of *xyn2* in *Hypocrea jecorina*. Eukariot Cell.

